# The Crosstalk between SARS-CoV-2 Infection and the RAA System in Essential Hypertension—Analyses Using Systems Approach

**DOI:** 10.3390/ijms221910518

**Published:** 2021-09-29

**Authors:** Dorota Formanowicz, Kaja Gutowska, Bartłomiej Szawulak, Piotr Formanowicz

**Affiliations:** 1 Department of Medical Chemistry and Laboratory Medicine, Poznan University of Medical Sciences, 60-806 Poznan, Poland; doforman@ump.edu.pl; 2 Institute of Computing Science, Poznan University of Technology, 60-965 Poznan, Poland; Kaja.Gutowska@cs.put.poznan.pl (K.G.); Bartlomiej.Szawulak@cs.put.poznan.pl (B.S.); 3 Institute of Bioorganic Chemistry, Polish Academy of Sciences, 61-704 Poznan, Poland

**Keywords:** SARS-CoV-2, essential hypertension, mathematical modeling, Petri nets

## Abstract

The severe acute respiratory syndrome coronavirus-2 (SARS-CoV-2), responsible for the coronavirus disease of 2019 (COVID-19) pandemic, has affected and continues to affect millions of people across the world. Patients with essential arterial hypertension and renal complications are at particular risk of the fatal course of this infection. In our study, we have modeled the selected processes in a patient with essential hypertension and chronic kidney disease (CKD) suffering from COVID-19, emphasizing the function of the renin-angiotensin-aldosterone (RAA) system. The model has been built in the language of Petri nets theory. Using the systems approach, we have analyzed how COVID-19 may affect the studied organism, and we have checked whether the administration of selected anti-hypertensive drugs (angiotensin-converting enzyme inhibitors (ACEIs) and/or angiotensin receptor blockers (ARBs)) may impact the severity of the infection. Besides, we have assessed whether these drugs effectively lower blood pressure in the case of SARS-CoV-2 infection affecting essential hypertensive patients. Our research has shown that neither the ACEIs nor the ARBs worsens the course infection. However, when assessing the treatment of hypertension in the active SARS-CoV-2 infection, we have observed that ARBs might not effectively reduce blood pressure; they may even have the slightly opposite effect. On the other hand, we have confirmed the effectiveness of arterial hypertension treatment in patients receiving ACEIs. Moreover, we have found that the simultaneous use of ARBs and ACEIs averages the effects of taking both drugs, thus leading to only a slight decrease in blood pressure. We are a way from suggesting that ARBs in all hypertensive patients with COVID-19 are ineffective, but we have shown that research in this area should still be continued.

## 1. Introduction

### 1.1. Research Context

SARS-CoV-2, responsible for COVID-19, has almost stopped the world [1,2,3,4,5]. Hence, for over a year, intensive research has been underway to understand the mechanisms of SARS-CoV-2 action and to find an effective treatment for it. Despite many peoples’ efforts, there is still no efficient antiviral therapy for SARS-CoV-2 infection, which frequently leads to fatal inflammatory responses and acute lung injury. SARS-CoV-2 turns out to be a “cunning criminal,” which binds to the membrane receptor—angiotensin converting enzyme 2 (ACE2)—and enters human cells. The ACE2 is not only the entry for SARS-CoV-2, it plays an important counter-regulatory role in the RAA system, promoting systemic vasodilatory and anti-inflammatory effects [6,7].

Since the dysfunction of the RAA system may be harmful to the cardiovascular system, additional confounding factors disrupting its balance, like SARS-CoV-2 infection, may prove fatal. This issue is critical because the RAA system helps to maintain a balance between vascular tone, blood pressure, and normal organ function in humans organisms. The RAA dysregulation has significant cardiovascular consequences [8].

In this study, the SARS-CoV-2 infection has been treated as an additional “signal” in a relatively unstable system, which is the patient’s body with essential hypertension and CKD [9]. So far, no studies have used such models to analyze the impact of SARS-CoV-2 on the hypertensive patient, which is much more prone than others to be severely affected by this harmful viral infection.

To better understand how SARS-CoV-2 impacts patients with essential hypertension and CKD, a group with a higher risk of adverse clinical outcomes and poor prognosis due to this viral infection, our recently published Petri net-based model [9,10] has been modified. The previous model focused primarily on the impact of innate, adaptive immunity and low-grade inflammatory processes on essential hypertension and CKD. To this model, the influence of SARS-CoV-2 has been added.

Why has a systems approach been used in this study? First, we should realize that every living organism is a complex system. Nowadays, this statement seems to be almost obvious, but its implications were not so evident in the not very distant past. Even today, it may not be clear that studying complex biological systems requires an application of appropriate analytical methods that differ from those traditionally used in medical sciences. It becomes more and more evident that for the structure and functionality of a living organism, not only are its elementary building blocks and their properties crucial, but also the interactions taking place among them. A viral infection that affects vital pathways in the organism leading to many “fatal disturbances” is a crucial example of a process that may be or even should be assessed by a systems approach. This means that the human organism needs to be taken into consideration as one large system of interactions between human cells and SARS-CoV-2 that forms a dense and complex network, which obviously influences the essential properties of the cells and tissues of an infected person.

However, until recently, and even now, a dominating approach for studying living objects is to focus on some of the elementary subunits composing them without taking into consideration the reach set of relationships between them and other parts of the investigated biological entity. This approach has been very successful and has resulted in many spectacular discoveries. However, it seems that there are severe limitations to it. As at least a significant part of the fundamental properties of living organisms results from the existence of the interaction networks, it is necessary to study these networks. Otherwise, it may be difficult or even impossible to go further towards our understanding of the nature of life.

Systems, not only the biological ones, should be studied using appropriate methods. Such methods have been developed for years in the area of systems sciences, and at least to some extent, they can be applied for studying living organisms. Such a systems oriented approach to studying biological phenomena gave rise to systems biology and systems medicine—interdisciplinary branches of science where living organisms are studied as complex biological systems. Here, important is the obvious fact that, in contrast to technical systems, they have not been developed by humans, so their exact structure has to be discovered.

The first and necessary step in studying a (biological) system is the construction of its model (see Section 2.1). The model should be as precise as possible since the precision, in many cases, determines the correctness of the model analysis results. Such a model should be expressed in a language of some branch of mathematics. In the case of biological phenomena, traditionally models based on differential equations have been formulated. But recently different types of models are often being developed, that is, those based on graph theory and related areas of mathematics.

Among them, the models expressed in the language of Petri nets theory, used in our studies, seem to be very promising. The models based on nets of this type are qualitative, which could be seen as a drawback, but in the context of biological systems, this is not the case. Indeed, a Petri net-based model can precisely describe the structure of the investigated system. This structure, in many cases, determines the functionality of the biological system. So, analyzing a Petri net-based model of such a system may result in a better understanding of the behavior of the latter. Besides, in the case of biological systems, quantitative data is often unavailable, or its availability is very limited. It causes severe problems for models based on differential equations since they are very sensitive to the parameter values. On the other hand, there are many extensions of Petri nets that allow for taking into account quantitative data of various types. So, it is possible to extend a qualitative Petri net-based model of a biological system when some qualitative data become available. It is worth mentioning that such an extension usually does not change the structure of the net being the model. Moreover, there are a lot of mathematical methods and software tools available for the analysis and simulation of Petri nets, which supports the study of the model. In addition, on one hand, a graphical representation of Petri nets is very intuitive and helps us to understand a structure of the modeled system, and on the other hand, as already mentioned, it can be analyzed using strict mathematical methods. Hence, choosing Petri nets for the modeling and analysis of such a complex biological phenomenon as SARS-CoV-2 infection in essential hypertensive patients with CKD is a good option.

### 1.2. Biological Background

The SARS-CoV-2 replicates in the upper respiratory tract, although in deeper areas of the lungs it can fuse with host cells and multiply by binding to the ACE-2 membrane receptor (angiotensin 2 converting enzyme; kininase II) due to the receptor-binding domain, encoded in the SARS-CoV-2 virus S (Spike) fusion protein.

Here, the mechanisms of the inflammatory process mediated by SARS-CoV-2 in a patient with essential hypertension accompanied by CKD are presented. Particular attention is paid to the issue of the RAA system; hence the detailed description of the functioning of this system and the relationship with other vital paths are provided here.

The RAA system involving the kidney, lungs, brain, and systemic vasculature is a hormone system that regulates blood volume and vascular resistance in the human organism. Dysregulation of the RAA system is a common feature of hypertension.

In our Petri net-based model, the RAA system acts through several steps to produce angiotensin II. It starts from prorenin synthesized by the kidney’s juxtaglomerular cells and angiotensinogen synthesized by the liver, through physiologically inactive angiotensin I, which is further converted by the angiotensin-converting enzyme (ACE), mainly synthesized by vascular endothelial cells in the lungs, to angiotensin II. Next, angiotensin II acts on the adrenal cortex to stimulate the release of aldosterone. Aldosterone acts on the cells collecting ducts in the nephron.

The angiotensin II, a multifunctional molecule, mediates many responses, mainly through angiotensin 1 and 2 receptors (AT1R and ATR2). These receptors belong to the superfamily of G protein-coupled receptors and play a vital role in maintaining cardiovascular homeostasis. In addition to this direct action, chronically elevated angiotensin II stimulates the production of reactive oxygen species, activation of the immune system, and changes in kidney dynamics, which have been shown to contribute to the development of hypertension, see [11,12].

In recent years, there is increasing evidence for the role of immunity and inflammation in the development of primary hypertension [13,14]. Hence, in the proposed model, a patient may develop essential hypertension through a few coexisting pathways:

(1) The dysregulated RAA system;
(2) The sodium balance disturbances (according to the new Tietze model [15]). Tietze has revealed that sodium, which is crucial for hypertension, can be osmotically immobilized by binding to polymerized glycosaminoglycans (GAGs) in the interstitial water space, and this is not related to water retention, see [16]. This sodium load causes an influx of mononuclear phagocyte system cells (MPS) into the interstitial water space. Here, MPS secrete a tonicity enhancer-binding protein (TonEBP), which, in turn, activates osmoprotective genes, including vascular endothelial growth factor (VEGF-C). The latter binds to the VEGFR3 receptor, stimulating lymphangiogenesis and lymphatic transport of the interstitial fluid [17]. It also binds to the VEGFR2 receptor and stimulates the synthesis of NO;
(3) The influence of immunological phenomena in the regulation of sodium balance and for the maintenance of angiotensin II-induced hypertension [18]. Since the Th17-IL-17 axis is believed to be hypertensive [19], the role of lymphocytes, mainly Th17, producing IL-17, in response to IL-23, as demonstrated in many autoimmune diseases, has been indicated in the model. Besides, the detrimental effect of other pro-inflammatory cytokines such as IFN-γ and TNF-α in the pathogenesis of hypertension has been stressed;
(4) An impact of the inflammatory processes (a role of the hypertension-specific neoantigens, newly formed antigens that have not been previously recognized by the immune system). The neoantigen is captured and presented by professional antigen presentation cells (APC), which induces the priming and activation of neoantigen-specific T cells in peripheral immune organs. The chronic exposure to exogenous activators (bacteria, viruses, diet, and airborne pollutants) and endogenous activators (bacteria, viruses, cellular contents released, and other molecules) may lead to inflammation-induced tissue injury in blood pressure-regulating organs. In people more susceptible to these factors, this can be sufficient to cause hypertension [14,20];
(5) The role of oxidative stress. A variety of enzymatic and non-enzymatic sources of reactive oxygen species exist in blood vessels [21]. The best-characterized source of reactive oxygen species (ROS) is NADPH oxidase and nitric oxide (NO) synthase, and they have been included in the model;
(6) The role of oxidative stress in CKD, see [22];
(7) Contribution of endothelial dysfunction, which is characterized by unbalanced vasodilation and contraction, increased ROS and pro-inflammatory factors, and a deficiency in the bioavailability of NO. The presence of endothelial dysfunction disrupts the permeability of the endothelial barrier, which is part of the inflammatory response in the development of cardiovascular disease, including hypertension, see [23].

The SARS-CoV-2 infection has been added to such an immune-inflammatory primary hypertension model. The virus in the model, like in the organism, replicates and multiplies by binding to the ACE2 membrane receptor (angiotensin 2 converting enzyme; kininase II) due to the receptor-binding domain encoded in SARS-CoV-2 virus S (Spike) fusion glycoprotein. So, SARS-CoV-2 uses ACE2 for entry, and the transmembrane protease serine 2 (TMPRSS2) for S protein priming [24]. As has been proven, ACE2 plays a vital role in the progression of SARS-CoV-2 infection [25,26].

ACE2 plays an essential role in the body [27]. It catalyzes the cleavage of angiotensin I into angiotensin 1-9 (ANG-(1-9)) and angiotensin II into angiotensin 1-7 (ANG-(1-7)), thus counteracting the effects of angiotensin II [28]. The balance between angiotensin II and ANG-(1-7) is critical in heart disease [29,30]. Angiotensin II binds to AT1R to cause vasoconstriction, whereas ANG-(1-7) elicits vasodilation mediated by AT2R. Besides, ANG-(1-7) is currently considered a biologically active member of the RAA, which, by acting through the Mas receptor, exerts an inhibitory effect on inflammation and vascular and cell growth mechanisms [31]. Numerous studies have shown a role of the ANG-(1-7)/ACE2/Mas axis in the evolution of hypertension, the regulation of renal function, and the progression of renal disease. Additionally, it has been suggested that a reduction in the activity of this axis may be a critical factor in the advancement of cardiovascular disease [32].

Animal studies have suggested that ACEIs, or sartans—angiotensin II receptor antagonists (ARBs)—can increase ACE2 expression [30,33], thereby increasing the availability of SARS-CoV-2 target molecules [34]. These studies have led to speculation that ACEIs and ARBs may prove harmful to COVID-19 patients [35,36,37,38].

Since then, the results of many observational studies have emerged that have provided data on whether ACEIs and ARBs are actually harmful in the context of the COVID-19 epidemic [39]. The message of these studies is consistent—they did not reveal any evidence of harm caused by continuous ACEIs and ARBs in COVID-19 patients, see [40,41,42,43,44,45].

Moreover, it has also been postulated in this clinical debate that increasing ACE2 levels may even improve clinical outcomes in patients with SARS-CoV-2 infection by protecting against lung injuries [46]. It should be noted that many of these studies are observational with a relatively short observation period.

On the other hand, there are significant studies that have strengthened professional society guidelines for not discontinuing ACEIs/ARBs treatment in COVID-19 patients when clinically indicated [47]. Thus, the treatment with ACEI and ARB drugs does not need to be altered for COVID-19 patients [48,49].

## 2. Results

### 2.1. Model

**Figure 1:**
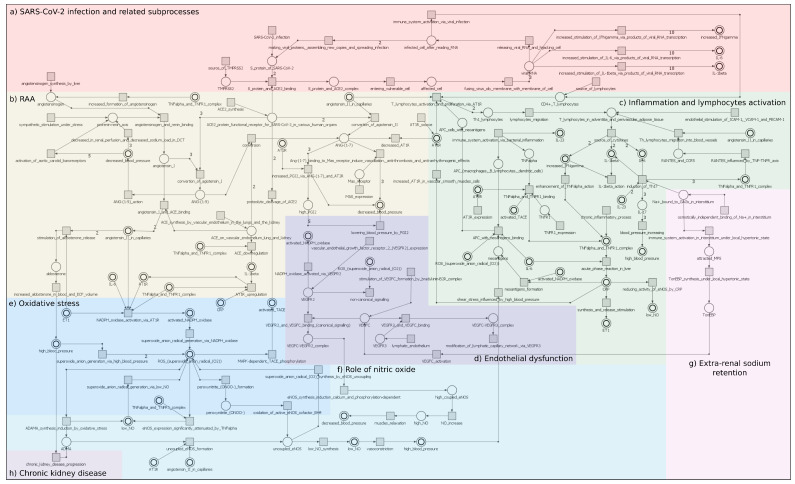
Model of the relation between SARS-CoV-2 infection and essential hypertension with division into particular modules.

The Petri net-based model of the relation between SARS-CoV-2 infection and essential hypertension (hereinafter referred to as the base model) has been created using a tool called Holmes [50]. The base model consists of 87 transitions (elementary processes), 55 places (biological components). All transition and place names are listed in Table 1 and Table 2. Furthermore, it is covered by 139,451 t-invariants. This is an unusually large number of t-invariants, which makes the analysis very difficult. Figure 1 shows the proposed model, taking into account division into particular modules: (a) SARS-CoV-2 infection and related subprocesses (orange); (b) RAA: renin–angiotensin–aldosterone system (yellow); (c) inflammation and lymphocytes activation (green); (d) endothelial dysfunction (dark purple); (e) oxidative stress (dark blue); (f) role of nitric oxide (light blue); (g) extra-renal sodium retention (pink); (h) chronic kidney disease (light purple). The described model is available in the Supplementary Materials.

In further analyses, apart from the base model, the base model with a knockout of SARS-CoV-2 and its related subprocesses has also been used. The model without viral infection consists of 76 transitions, 49 places and it is covered by 25,997 t-invariants. This model is reduced by five places ($p_{40}$, $p_{41}$, $p_{42}$, $p_{44}$, $p_{45}$, $p_{50}$) and 11 transitions ($t_{57}$, $t_{58}$, $t_{59}$, $t_{61}$, $t_{64}$, $t_{68}$, $t_{76}$, $t_{78}$, $t_{84}$, $t_{85}$, $t_{86}$), which are marked in bold fonts in Table 1 and Table 2.

### 2.2. Analysis

#### 2.2.1. Significance Analysis for the Model with SARS-CoV-2 Taking into Account All t-Invariants and Only Those Related to SARS-CoV-2

The significance analysis as a structural analysis is based on t-invariants. It allows for the identification of essential elementary processes modeled by single transitions. The significance analysis has been performed on two levels. The first one concerns all t-invariants of the base model, which is focused on the relation between SARS-CoV-2 infection and essential hypertension. The second one concerns only selected t-invariants of the base model, which are related to the SARS-CoV-2 module (the number of such t-invariants is equal to 113,454, which represent approximately 81% of all t-invariants in the base Petri net model). t-invariants associated with the SARS-CoV-2 module are understood as only those which in their supports contain at least one transition from the SARS-CoV-2 module, that is, ($t_{57}$, $t_{58}$, $t_{59}$, $t_{61}$, $t_{64}$, $t_{68}$, $t_{76}$, $t_{78}$, $t_{84}$, $t_{85}$, $t_{86}$). t-invariants associated with the SARS-CoV-2 module (113,454) were identified by subtracting the set of t-invariants of the model with the knockout of the SARS-CoV-2 module (25,997 t-invariants) from the set of t-invariants of the base model (139,451 t-invariants). The results of the significance analysis for selected elementary processes (those for which the significance is above 50%) for all t-invariants and t-invariants associated with the viral module are presented in Table 3. This table contains two main columns: “occurrence frequency of transitions in all t-invariants of the model with SARS-CoV-2 (139,451 t-inv.)” and “occurrence frequency of transitions in selected t-invariants of the model with SARS-CoV-2—only those related to the SARS-CoV-2 module (113,454 t-inv.)", where information about the frequency of a given transition in support of t-invariants and its percentage contribution in all supports of t-invariants is included. For each transition its number (column “ID”), name (column “name of transition”) and the module in which it occurs (column “modules”) are given. Column “modules” corresponds to the modules in Figure 1. For example, transition $t_{0}$, which corresponds to the elementary process called “neoantigen formation”, is assigned to the inflammation and lymphocytes activation module, which is marked as block c in Figure 1. The frequency of transitions $t_{0}$ is equal to 84,808, which means that it occurs in exactly 84,808 supports of t-invariants out of 139,451 (all t-invariants); thus, its percentage contribution (significance) is equal to 60.82%. Moreover, the frequency of this transition only in supports of t-invariants associated with the viral module is 70,158 out of 113,454 (only t-invariants associated with the virus); thus, the percentage is equal to 61.84%.

The significance analysis for the model of the relation between SARS-CoV-2 infection and essential hypertension (for all 139,451 t-invariants) has shown that the most crucial elementary reactions are mainly included in RAA, inflammation and lymphocytes activation, and SARS-CoV-2 infection modules. Nevertheless, there are also significant elementary processes from other modules, such as oxidative stress, extra-renal sodium retention, engagement of NO and endothelial damage.

**Figure 2:**
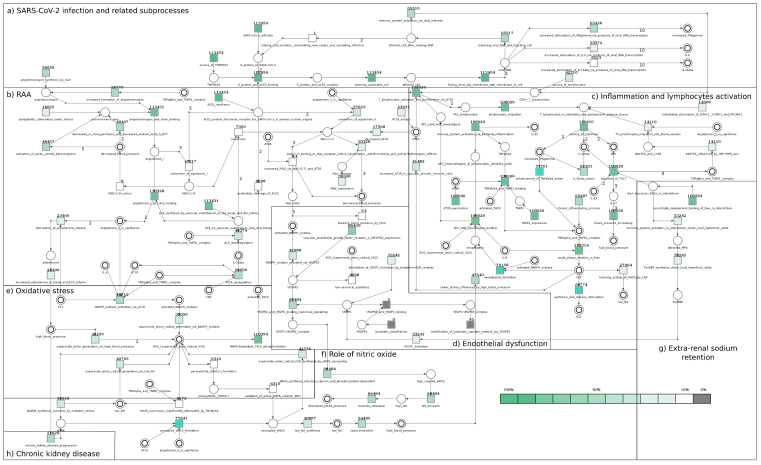
Significance analysis for each transition performed only for SARS-CoV-2 related t-invariants. Transitions are marked in a color gradient from white to different green saturations (if the color has a higher intensity, the given elementary process has greater significance) or they can be marked with a grey color (if transitions are not related to the t-invariants associated with the viral module).

The significance analysis performed only for t-invariants related to SARS-CoV-2 has allowed the determination of how crucial the transitions corresponding to elementary processes are in these selected t-invariants. On this basis it has also been possible to find out which elementary processes are independent of the virus module. Table 3 includes a column called “occurrence frequency of transitions in selected t-invariants of the model with SARS-CoV-2—only those related to the SARS-CoV-2 module (113,454 t-inv.)”, which is complemented by Figure 2. This figure illustrates the significance of each transition in the proposed model. Transitions are marked in a color gradient from white to different saturations of green (if the color has a higher intensity, the given elementary process has greater significance), or they can be marked with a grey color (if transitions are not related to the t-invariants associated with the viral module).

Based on the above analysis, both for all t-invariants and for only SARS-CoV-2 related t-invariants, it can be seen that the significances for each transition is similar. This is because t-invariants associated with the SARS-CoV-2 module consist of the majority of all t-invariants (approximately 81%). Table 3 contains only those transitions for which a significance is above 50% in both the analysis of all t-invariants and those t-invariants only associated with the SARS-CoV-2 module. This analysis, apart from finding essential elementary processes, has also allowed us to find irrelevant ones or not related to SARS-CoV-2. For example, the analysis has allowed us to notice that there are three elementary processes, that is, the modification of the lymphatic capillary network via VEGFR3 ($t_{13}$), VEGFR3 and VEGFC binding ($t_{17}$), and the lymphatic endothelium ($t_{20}$) belonging to the endothelial damage module, which are not associated with the virus t-invariants. Thus, these processes work independently of the SARS-CoV-2 virus in the proposed model.

A closer look at the results obtained in this analysis is presented in the next section.

#### 2.2.2. Comparison of the Significance between the Models with and without SARS-CoV-2

As well as the significance analysis for the model with SARS-CoV-2, we use the same analysis for the model with the knockout of SARS-CoV-2 and reactions directly related to the viral infection have been performed. The comparison results for the significance analysis of the model of essential hypertension with and without the presence of SARS-CoV-2 are presented in Table 4. This table contains columns called “the model with SARS-CoV-2 infection (139,451 t-inv.) frequency trans./t-inv.” and “the model without SARS-CoV-2 infection (25,997 t-inv.) frequency trans./t-inv.” which contain information about the occurrence frequency of a given transition in supports of t-invariants and a percentage ratio in the context of all t-invariants. The column named “difference in p.p.” contains differences in significance (expressed as percentage points) for given elementary processes in the comparison between the model containing SARS-CoV-2 and the model without the virus.

A summary of the most significant differences obtained during the comparative analysis is included in the Table 5.

It should be realized that the significance analysis is an analysis at the level of elementary processes (transitions) that are components of larger subsystems (biological modules). Some elementary processes may be more or less significant in a particular module, which is generally considered essential for the functioning of the whole system.

#### 2.2.3. The Analysis of MCT Sets

Analysis of Maximal Common Transition sets (MCT sets) relies on the determination of a biological context for some functional blocks [51,52]. MCT sets contain transitions, which are elements of supports of exactly the same t-invariants (cf. [53]). The proposed Petri net-based model of the relation between SARS-CoV-2 infection and essential hypertension is divided into 10 MCT sets. MCT sets corresponding to some non-trivial functional blocks are described in Table 6.

A similar analysis of MCT sets has been performed for t-invariants solely associated with the SARS-CoV-2 virus. Most of the MCT sets show no changes in comparison to MCT sets calculated on the basis of all t-invariants. However, there is a difference in one of them, that is, $m_{2}$ contains an additional transition $t_{63}$, which does not change the overall biological meaning of this MCT set. Obviously, the MCT set containing virus-independent transitions cannot be determined for t-invariants solely associated with the SARS-CoV-2 module. Therefore, $m_{5}$ is not present in MCT sets based on viral t-invariants.

**Figure 3:**
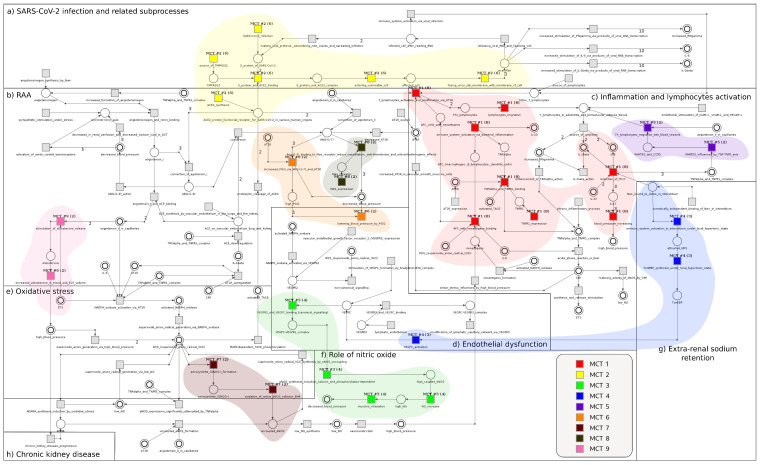
A graphical representation of the MCT sets for the t-invariants solely associated with SARS-CoV-2 virus.

A graphical representation of the MCT sets for the viral t-invariants is shown in Figure 3. MCT sets for viral t-invariants have been analyzed in terms of the significance of particular transitions contained in these sets. Among all MCT sets, set $m_{1}$ contains the most significant transitions; the occurrence frequency in SARS-CoV-2-related t-invariants is approximately 95%. This set ($m_{1}$) is associated with some transitions of the inflammation and lymphocytes migration module, which include, among others, $t_{23}$, which corresponds to TNFR1 expression, and $t_{22}$, which corresponds to TNF-α and TNFR1 binding.

As well as non-trivial sets containing transitions of high significance, there are trivial MCT sets, that is, single transitions. The significance analysis for each transition is included in Table 3 for both all t-invariants and those associated only with the SARS-CoV-2 module.

#### 2.2.4. The Knockout Simulation

Three different knockout simulations that are mainly focused on the RAA module have been performed. They simulate the hypertensive patient organism receiving selected medications: (1) ACEIs, which are one of four drug classes recommended for initial therapy; (2) sartans—ARBs (formally AT1R blockers); and (3) ACEIs and ARBs simultaneously. Both of these medications are the first-line drugs in the management of hypertensive patients.

As a result of these knockout analyses, data about the model’s behavior, when selected elementary processes were excluded, have been collected. More precisely, the average number of firings of the elementary process (transition) in 10,000 simulation steps averaged over all 100 performed repetitions have been obtained. These results have been analyzed in the context of the influence on blood pressure regulation and the development of SARS-CoV-2 infection.

In the first simulation, a hypothetical patient with essential hypertension and CKD has been medicated by ACEIs. Therefore, elementary processes related to ACE have been knocked out. This knockout has been focused on elementary processes, which are ACE synthesis by vascular endothelium in lungs and kidney (transition $t_{46}$) and ACE downregulation (transition $t_{51}$). As a result of the knockout simulation of these transitions, all subprocesses directly related to ACE and angiotensin II have been excluded.

The excluded elementary processes were, among others, NADPH oxidase activation, uncoupled eNOS formation, T lymphocytes activation and proliferation, Th lymphocytes migration into blood vessels, but also elementary processes, such as the conversion of angiotensin I to angiotensin II by ACE, conversion of ANG-(1-9) to ANG-(1-7) by ACE, conversion of angiotensin II to ANG-(1-7), or the stimulation of aldosterone release. The next step assesses whether blocking ACE reduced blood pressure. Therefore, the average number of firings of transitions associated with blood pressure increases compared to the reference set (a situation in which a patient suffering from essential hypertension does not receive any blood pressure medications) has been analyzed. The analysis of the firing of transitions associated with an increase of blood pressure, such as vasoconstriction or blood pressure increasing via IL-17, has shown the expected decrease in the frequency of transitions firing, which corresponded to lowering blood pressure after ACEIs treatment.

Next, the analysis of whether ACEIs used for hypertension treatment could increase ACE2 expression and thus contribute to enhancing the development of viral infection by allowing SARS-CoV-2 to more intensely enter vulnerable cells has been performed. However, in this case, no frequency changes in firing transitions related to the synthesis of ACE2 and the binding of S protein and ACE2 were observed. In conclusion, this simulation has shown that the use of ACEIs does not increase the development of SARS-CoV-2 infection in hypertensive patients with coexisting CKD.

In the second simulation, a patient with essential hypertension and CKD received sartans (ARBs). Therefore, elementary processes related to AT1R were knocked out. This knockout has been focused on: increased AT1R in vascular smooth muscles cells (transition $t_{6}$), AT1R source (transition $t_{7}$), decreased AT1R (transition $t_{66}$), and AT1R upregulation (transition $t_{81}$). As a result of the knockout simulation of these transitions, all subprocesses directly related to AT1R have been excluded. The excluded elementary processes were, among others, NADPH oxidase activation, uncoupled eNOS formation, and T lymphocytes activation and proliferation. Apart from excluding these transitions, we have been interested in other elementary processes, such as converting angiotensin II to ANG-(1-7) and the stimulation of aldosterone release.

In these reactions, the expected increase in the frequency of transition firing has been observed. Similar to the first knockout simulation, we assessed whether blocking AT1R reduced blood pressure. Therefore, the average number of firings of transitions associated with blood pressure increases compared to the reference set (a situation in which a patient suffering from essential hypertension does not receive any blood pressure medications) has been analyzed. The analysis of the firings of transitions associated with an increase of blood pressure has not shown the expected decrease. This analysis has shown an increase in the average number of firings of these transitions, which means an increase in blood pressure despite taking sartans. Hence, it seems that in the case of SARS-CoV-2 infection in patients with coexisting disorders such as essential hypertension and CKD, physicians should consider, if it is possible, not prescribing AT1R blockers for the treatment of hypertension. Based on the systems analyses performed, it seems that these drugs may not be sufficiently effective.

An analysis of whether sartans used in the treatment of hypertension could increase ACE2 expression and thus contribute to increasing the development of viral infection by allowing SARS-CoV-2 to more intensely enter vulnerable cells has also been conducted. In the case of ACEIs treatment, no change in the frequency of the firing of transitions related to ACE2 synthesis and the binding of S protein and ACE2 has been observed. In conclusion, this simulation has shown that the treatment with ARBs does not increase the development of SARS-CoV-2 infection.

The third simulation concerned a patient receiving both ACEIs and ARBs. Therefore, elementary processes related to ACE and AT1R have been knocked out. This knockout was focused on elementary processes which are as follows: increased AT1R in vascular smooth muscle cells (transition $t_{6}$), AT1R source (transition $t_{7}$), ACE synthesis by vascular endothelium in lungs and kidneys (transition $t_{46}$), ACE downregulation (transition $t_{51}$), decreased AT1R (transition $t_{66}$), and AT1R upregulation (transition $t_{81}$). The mentioned transitions that have been excluded in the third simulation were the same as those in the first and second simulations. Such a set of transitions can be obtained as the sum of the sets of transitions from the first and the second simulations. Thus, as a result of the knockout of such a set of transitions, exactly the same elementary processes as in the previous simulations have been excluded. To be more precise, the elementary processes which have been excluded in the third simulation are the sum of the elementary processes excluded during the first and the second simulations. All transitions excluded as a consequence of this knockout correspond to subprocesses directly related to ACE and AT1R.

The performed knockout allowed the determination of whether blocking ACE and AT1R really decreases blood pressure. Therefore, similarly to the first and second simulations, the average number of firings of transitions associated with blood pressure increase has been analyzed. As a result of this simulation, a slight decrease in the frequency of the firing of transitions related to the increase of blood pressure has been observed. However, it should be noted that both ACE and AT1R have been blocked simultaneously, so a certain average of the described earlier effects has been observed. To be more precise, a slight decrease of blood pressure has been observed, which is more beneficial for the hypothetical patient compared to the results of the second simulation (where the use of ARBs has not caused a decrease of blood pressure, and may even cause a slight increase). The described decrease of blood pressure is, however, definitely less significant or even insignificant in comparison to the use of ACEIs. Therefore, it seems that the simultaneous use of angiotensin-converting enzyme inhibitors and angiotensin receptor blockers may not act as expected—they do not significantly reduce blood pressure in patients suffering from COVID-19.

As in the previous simulations, it has also been assessed whether the simultaneous use of ACEIs and ARBs could increase ACE2 expression and thus contribute to increasing the development of SARS-CoV-2 infection. However, in the case of this simulation, as in the first and in the second simulations, no change in the frequency of the firing of transitions related to the synthesis of ACE2 and the binding of S protein and ACE2 has been observed. In conclusion, this simulation has shown that the use of angiotensin-converting enzyme inhibitors and angiotensin receptor blockers simultaneously do not contribute to increasing the development of SARS-CoV-2 infection.

## 3. Discussion

To determine how SARS-CoV-2 may affect the modeled system, that is, a patient suffering from essential hypertension with CKD, a significance analysis between both the models with and without the influence of SARS-CoV-2 has been performed. These analyses have been based on the structure of the Petri net model and the significance of individual elementary processes (transitions). This means that the importance of elementary processes can increase or decrease in the presence of the virus. The results of this analysis are described in more detail below.

The SARS-CoV-2 module is present only in the model with a virus infection. Therefore, Table 4 does not include a comparison for these transitions: $t_{57}$—S protein and ACE2 binding, $t_{58}$—entering vulnerable cell, $t_{59}$—fusing virus membrane with membrane of cell, $t_{61}$—SARS-CoV-2 infection, $t_{64}$—releasing viral RNA and hijacking cell, $t_{68}$—making viral proteins, assembling new copies and spreading infection, $t_{76}$—source of TMPRSS2, $t_{78}$—immune system activation via viral infection, $t_{84}$—increased stimulation of IFNγ via products of viral RNA transcription, $t_{85}$—increased stimulation of IL-1β via products of viral RNA, $t_{86}$—increased stimulation of IL-6 via products of viral RNA transcription. The viral infection module, in general, is characterized by a high significance. However, if each particular elementary process is considered, rather than an entire module, it can be noticed that some elementary processes are more or less important than others. The most significant elementary processes concern entry of the virus and viral infection. In addition to these processes, there are slightly less significant but still important elementary processes that are additionally stimulated by the presence of SRAS-CoV-2, that is, increased stimulation of IFN-γ (central antiviral mediator), increased stimulation of IL-1β, and increased stimulation of IL-6. It is interesting that the stimulation of IFN-γ is the most significant (46.92%), followed by IL-6 (significance is 24.07%), while the IL-1β (6.81%) stimulation is the least significant.

Moreover, another essential cytokine, TNF-α, appearing in the model as a component of the inflammation and lymphocytes activation module (see the biological modules in Table 4), has been analyzed. TNF-α significance is higher in the model with the SARS-CoV-2 virus (the significance is equal to 65.96%, increased by 11.26 p.p. compared to the model without the virus). As described above, additional cytokine stimulation in the model with the SARS-CoV-2 virus is directly related to the cytokine storm. The cytokine storm is related to the overproduction of inflammatory cytokines with a wide range of biological activities from various tissues and cells. These cytokines give positive feedback to other immune cells and lead to further recruitment to sites of inflammation [54].

For all modules except the viral module, a comparison of the significance analysis for the model of the relation between SARS-CoV-2 infection and essential hypertension versus the model of essential hypertension without SARS-CoV-2 module has been performed (analysis results are included in Table 4). Significant differences have been observed only in the case of two modules, that is, the inflammation and lymphocytes activation module and the RAA module, described below.

In the inflammation and lymphocytes activation module, most of the elementary processes are characterized by significant differences, which were both positive and negative.

- Significant positive differences mean that the significance for given elementary processes is greater in the model with the SARS-CoV-2 virus. The following transitions indicating positive differences have been distinguished:
  - The enhancement of TNF-α action (11.26 p.p.), TNF-α and TNFR1 binding (5.47 p.p.) and TNFR1 expression (5.47 p.p.) (see Table 4)—TNF-α is one of the cytokines involved in the cytokine storm, so the increase in its significance in the model with the virus is expected (which was also mentioned at the beginning of Section 3).
  - The immune system activation via bacterial inflammation (5.47 p.p.), and APC with neoantigens binding (5.47 p.p.)—the significance increase of these elementary processes in the model with the virus is not entirely intuitive. However, when a virus enters the host cells, the immune system (which is essential for immune response) is stimulated. This leads to an enhancement of the chronic bacterial process that is present before the SARS-CoV-2 infection. Activating the immune response is a tightly regulated, coordinated effort, the purpose of which is to control and eliminate exogenous microorganisms while responding to endogenous ligands. Establishing the proper balance of inflammation is crucial because chronic inflammatory processes lead to various host pathologies. Bacterial pathogens can induce chronic inflammation through a wide variety of evolved avoidance strategies that interfere with immune regulation [55]. The appearance of SARS-CoV-2 additionally disturbs this system and is most likely the reason for the intensification of the bacterial processes underlying essential hypertension.
  - T lymphocytes activation and proliferation via AT1R (5.47 p.p.), induction of Th17 (5.47 p.p.), and lymphocytes migration (5.47 p.p.)—the positive difference thus significance increase in the model with SARS-CoV-2 virus for these elementary processes is warranted, because an immune system response is the first line of defense occurring during viral infection.
  - Blood pressure increasing (5.47 p.p.)—this elementary process is more significant in the model with the virus. SARS-CoV-2 infection may downregulate ACE2 levels on the cell surface, leading to a decrease in ACE2 activity in the infected organs. Lowering ACE2 levels in response to SARS-CoV2 binding may serve as a mechanism to counteract viral infection at the expense of increasing angiotensin II. The presence of the virus induces ACE2 downregulation and thus contributes to an increase in blood pressure. It should be noticed that the model corresponds to a patient who, apart from SARS-CoV-2 infection, additionally suffers from accompanying disorders such as essential hypertension and CKD.
  All elementary processes listed above with a positive difference equal to 5.47 p.p. occur in exactly the same number of t-invariant supports (131,032). Therefore, the calculated difference is the same for all the mentioned processes. Moreover, transitions corresponding to these elementary processes belong to the same MCT set $m_{1}$. The exception is the elementary process “The enhancement of TNF-α” which occurs in a smaller number of t-invariant supports (91,981). This process, although it occurs in a smaller number of t-invariant supports, shows a greater positive difference than other elementary processes from the same module. It can be explained by the fact that this process is directly related to the IFNγ cytokine, which is additionally stimulated by the SARS-CoV-2 module. The other mentioned elementary processes are not directly stimulated by the viral module.
- Significant negative differences mean that the significance for given elementary processes is greater in the model without SARS-CoV-2 virus. In the case of the presence of the virus, the action of these processes may be weakened by other more significant processes, which is why their importance is decreased. The following transitions characterized by negative differences have been distinguished:
  - Source of lymphocytes (−41.51 p.p.)—the source of lymphocytes in general context activates the immune response, which is not solely dependent on the presence of the SARS-CoV-2 virus. This transition has been modeled as an additional source of lymphocytes that can be activated independently of SARS-CoV-2. Hence, as expected, this elementary process is definitely less important in the model with the virus.
  - Chronic inflammatory process (−37.73 p.p.)—it is counterintuitive that the chronic inflammation process that stimulates TNF-α cytokine is characterized by negative differences. This means that this particular elementary process is less significant in the model with the SARS-CoV-2 virus in comparison to the model without SARS-CoV-2. This is unsubstantiated, especially since a positive difference has been shown (which means greater significance in the model with the virus) for the elementary process corresponding to the enhancement of this cytokine (positive differences for the enhancement of TNF-α action have been described above). However, it should be noted that the modeled system is in a state of chronic inflammation even before the appearance of the virus. In the model without the virus the inflammatory process underlying hypertension is one of the key factors in maintaining essential hypertension. Therefore, its significance is high (99.51%). On the other hand, entry of the virus into the cells of a patient with persistent essential hypertension (which is sustained by a chronic inflammatory process) may result in this inflammatory process; although it is important, it loses its importance. This can be due to the entry of the SARS-CoV-2 into the patient’s cells being the additional strong influencing factor.
  - Th lymphocytes migration into blood vessels (−5.75 p.p.), RANTES influenced by TNF-TNFR axis (RANTES belong to the cytokines activating T cells) (−5.75 p.p.), endothelial stimulation of ICAM-1, VCAM-1 and PECAM-1 (elementary process responsible for the stimulation of T lymphocytes) (−5.50 p.p.)—these elementary processes are important components of the immune system response and therefore it seems that they should indicate positive differences, not negative ones (indicating that their significance is lower in the model with SARS-CoV-2). It may be related to the fact that in patients with a severe case of COVID-19, a decrease in lymphocytes is observed [56].
  It can be noticed that the greater the negative difference, the more a given elementary process is independent of the viral module. Both the elementary processes, that is, “Source of lymphocytes” and “Chronic inflammatory process”, occur and are relevant in the model without SARS-CoV-2 infection. Moreover, due to the coexistence of other disease entities in the proposed model, such as CKD or essential hypertension, the inflammatory process is constantly maintained.

In the RAA module (unlike in the inflammation and lymphocytes activation module), most of the elementary processes do not change their significance. It can be assumed that these reactions occur with the same intensity regardless of the presence of the virus. Nevertheless, there are some important differences to note.

- Significant positive differences mean that the significance of given elementary processes is greater in the model with the SARS-CoV-2. We have distinguished the following transitions characterized by positive differences:
  - ACE2 synthesis (44.82 p.p.)—ACE2 plays the role of a functional host receptor for SARS-CoV-2. Increased expression of ACE2 may lead to an increased susceptibility to SARS-CoV-2 entering into host cells. Higher significance in the model with the virus is expected.
  - AT2R expression (5.39 p.p.)—AT2R is an anti-inflammatory receptor and appears in response to the presence of a viral infection. Higher significance in the model with the virus is expected.
- Significant negative differences mean that the significance of given elementary processes is greater in the model without the SARS-CoV-2. In the case of the presence of the virus, the action of these subprocesses may be covered up by other more significant subprocesses; that is why their importance is decreased. The following transitions characterized by negative differences have been distinguished:
  - ACE downregulation (−19.34 p.p.)—ACE2 is downregulated via the presence of SARS-CoV-2. Therefore, the balance between ACE and ACE2 is disturbed (which leads to an increase of ACE levels). The elementary processes such as ACE downregulation indicate higher significance in the model without the virus, which is the expected result.
  - Increased AT1R in vascular smooth muscles cells difference (−7.69 p.p.), and AT1R source (−5.17 p.p.)—it seems that AT1R, as a pro-inflammatory receptor, should indicate a higher significance in the model with SARS-CoV-2, but the result is the opposite. The knockout simulation of AT1R (simulation of receiving AR1R blockers) determined that there is no decrease in blood pressure. This leads to the assumption that this antihypertensive medication does not work properly in COVID-19 patients. However, this applies to ARBs rather than ACEIs. The antihypertensive action of AT1 antagonist may in part be due to increased Ang II metabolism by ACE2. So if there is SARS-CoV-2 infection, the availability of ACE2 becomes lower. Hence, the increased metabolism of Ang II does not take place, which in turn causes an increase in the concentration of Ang II and may even cause an increase in blood pressure. The conclusions of this analysis have been summarized below.

In addition to the comparative analyses of the model with SARS-CoV-2 and the model without the virus, knockout simulations on the first of them have been performed. More specifically, the simulations have been performed on the model with SARS-CoV-2 because it describes a hypothetical patient with essential hypertension and CKD, infected with the SARS-CoV-2. Using this model, three situations in which patients received ACEIs inhibitors, ARBs, or both ACEIs and ARBs simultaneously have been simulated.

The results of the ACE blocking simulation have confirmed that taking ACE inhibitors decreases blood pressure, which confirms that the model is correct. However, an increased expression of the ACE2 (which may be induced by medications used in the treatment of essential hypertension) has not been observed and thus increased development of SARS-CoV-2 infection has not been noticed. In conclusion, it appears that ACEIs can be used in COVID-19 without significant risk. These drugs have properly reduced the increased blood pressure in hypertensive patients with SARS-CoV-2 infection.

The results of AT1R blocking simulation demonstrate that taking ARBs does not decrease blood pressure in SARS-CoV-2 infected hypertensive patients. They even lead to a slight increase in blood pressure. As in the case of the first simulation, an increased expression of ACE2 has not been observed and, thus, increased development of SARS-CoV-2 infection has not been noticed. In conclusion, it seems that ARBs may not act properly (they do not lower blood pressure in the model) in patients with COVID-19.

The results of simulating the simultaneous blocking of ACE and AT1R demonstrate that taking both drugs (ACEIs and ARBs) does not significantly reduce blood pressure. Only a slight decrease in blood pressure, possibly even insignificant, has been observed. As in the first simulation—ACE blocking and the second simulation—AT1R blocking, no increased ACE2 expression and thus no increased development of SARS-CoV-2 infection has been observed. It seems that the simultaneous use of angiotensin-converting enzyme inhibitors and angiotensin receptor blockers may not act properly, that is, they do not significantly reduce blood pressure in patients with SARS-CoV-2 infection.

## 4. Materials and Methods

Petri nets are mathematical objects which can be used for modeling concurrent systems of many types. However, for years they have been used mainly in the area of technical systems, especially computer systems. In the last two decades there is a growing interest in applying Petri nets for the modeling and analysis of complex biological systems [57].

A Petri net has a structure of a directed weighted bipartite graph. This means that it is composed of vertices of two types called places and transitions. Some of the vertices can be connected by an arc but only vertices of different types can be connected in this way. When a Petri net is a model of some system, places usually correspond to passive elementary components of the system (such as chemical compounds) while transitions correspond to active components (such as chemical reactions). Arcs describe causal relations between passive and active components.

As mentioned before, the directed bipartite graph is a structure of a Petri net but there is one more important type of Petri net component, that is, tokens. They reside in places and flow from one place to another through transitions. The flow of tokens corresponds to a flow of substances, information and so forth in the modeled system. It is governed by a rule of transition firing. Each arc is labeled by a positive integer called weight, which determines a number of tokens flowing along the arc. The places that directly precede a given transition are pre-places of this transition, while the places that directly follow the transition are its post-places. A transition is active if in each of its pre-places the number of tokens is equal to or greater than the weight of an arc connecting the place with the transition. An active transition can be fired, which means that tokens flow from its pre-places to its post-places and the numbers of flowing tokens are equal to the weights of the respective arcs. There is an important exception to this rule. If a transition does not have pre-places (i.e., there are no arcs incoming to this transition), it is always active (i.e., it can be fired at every moment). Such transitions are called input transitions and they often represent the sources of some signals entering the modeled system from its environment. Firing a transition means that the elementary process modeled by it takes place in the system.

Petri nets have a very intuitive graphical representation. In this representation, places are depicted as circles, transitions as rectangles or bars, arcs as arrows, tokens as dots or integer numbers residing within places, and weights as integer numbers located above arcs (if a weight is equal to one, it is not represented, for simplicity). It is possible to represent a given place by more than one circle labeled by the same name of the place. Such multiple representations are called logical places and they are used to simplify a scheme of the net in the case of nets containing great numbers of places, transitions and arcs [53,57,58,59].

Petri nets have many properties that can be considered during the analysis of the modeled system. In the case of biological systems, especially important is the analysis based on transition invariants (called t-invariants). An invariant of this type is vector *x*, being a solution of the matrix equation:

(1) A·x=0,

where *A* is an incidence matrix of the net. In this matrix, columns correspond to transitions while rows correspond to places. Entry $a_{ij}$ of matrix *A* is a number equal to a difference between numbers of tokens residing in place $p_{i}$ before and after firing transition $t_{j}$. The length of vector *x* is equal to the number of transitions in the net and entry $x_{j}$ of this vector corresponds to transition $t_{j}$. Every t-invariant *x* has support s(x), which is a set of transitions corresponding to positive entries of *x*. More formally, $s\left(x\right)=\left\{t_{j}:x_{j}>0,j=1,2,\ldots ,m\right\}$, where *m* is the number of transitions.

If every transition from support s(x) is fired $x_{j}$ times, then a distribution of tokens over the set of places (called a marking of the net) remains unchanged. Since a marking of a net corresponds to a state of the modeled system, it means that t-invariants are counterparts of subprocesses which do not change a state of the system. It makes them especially important in the analysis of models of biological systems [53].

t-invariants can be a basis for grouping transitions into MCT sets [51,52]. A set of this type contains transitions which are elements of supports of exactly the same t-invariants. From this follows that the sets are disjoint and correspond to some subnets of the net. If the net is covered by t-invariants, that is, if every transition belongs to a support of at least one t-invariant, each transition is an element of exactly one MCT set. However, it may happen that some of them are single element sets—they are called trivial MCT sets. Those MCT sets which are not the trivial ones usually correspond to some functional modules of the modeled biological system [53,57,60].

Apart from the analysis of MCT sets, there are other methods of analysis of Petri net-based models, for example, the significance analysis, the knockout analysis and the knockout simulation [61,62]. It should be noted that the knockout analysis and the knockout simulation are two different methods of knockouts.

The significance analysis is based on the occurrence frequency of each transition in all supports of t-invariants. Since transitions correspond to elementary processes, such an analysis allows us to distinguish the most significant elementary processes, but also irrelevant ones. As a result of the significance analysis information about the frequency of a given transition in supports of t-invariants and its percentage contribution in all supports of t-invariants is obtained. To complement the significance analysis, a knockout analysis also can be performed.

The knockout analysis allows the exclusion of selected transitions or sets of transitions corresponding to some subprocesses or whole biological modules. After the knockout of selected transitions, a current number of t-invariants can be determined. On this basis, it can be indicated how many subprocesses have been excluded in consequence of knockout, which in turn allows the estimation of how crucial the selected elementary processes or whole subprocesses are. Based on significance and knockout analyses one can perform a comparative analysis for the base model and the model with a knockout of a particular selected module. This comparison allows the estimation of how an excluded module affects the whole modeled system and whether it stimulates or inhibits other subprocesses or is independent of different subprocesses and does not affect them in any way.

As a result of the comparative significance analysis besides the occurrence frequency of a given transition in all supports of t-invariants and a percentage ratio in the context of all t-invariants, differences in significance (expressed as percentage points) are also obtained. These differences can be divided into three categories:

- insignificant difference: −5 p.p. < difference < 5 p.p.
- significant positive difference: difference ≥ 5 p.p.
- significant negative difference: difference ≤−5 p.p.

**Figure 4:**
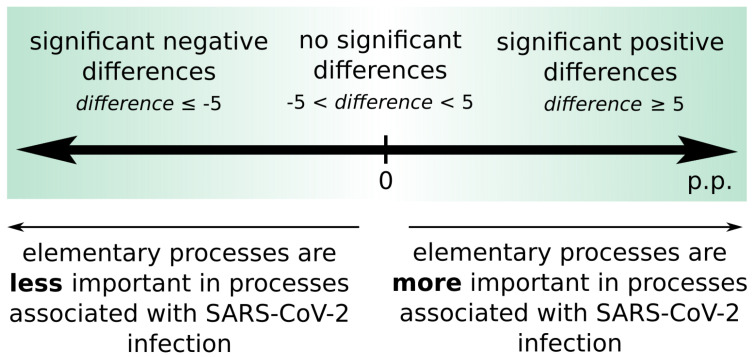
Interpretation of the positive and negative differences including their significance.

A transition characterized by significant positive difference means that the significance of an elementary process corresponding to it is greater in the model with SARS-CoV-2 infection in comparison to the model without the virus. On the other hand, a transition characterized by significant negative differences means that the significance of an elementary process corresponding to it is smaller in the model with SARS-CoV-2 infection in comparison to the model without the virus. It may be related to the fact that, in the case of the presence of the virus, such a process may be covered up by other more significant subprocesses, that is why their importance is decreased. Importantly, the greater the positive difference, the more a given elementary process is associated with the viral module (it may be directly stimulated by the SARS-CoV-2 infection), while the greater the negative difference, the elementary process is more independent of the viral module. The described relations are shown in Figure 4.

In addition to the knockout analysis, which concerns the structure of the Petri net model, the second method of knockout called a knockout simulation has also been performed. The purpose of the knockout simulation is to collect data about the behavior of the Petri net model in the case of excluding selected transitions. As a result, an average number of firing transitions in all simulation steps averaged over all performed simulations can be obtained. A performed simulation with a knockout (knockout dataset) can be compared to a simulation of a base model without a knockout (reference dataset). On this basis, an increase or decrease in an average number of the firings of a given elementary process can be observed.

## 5. Conclusions

Since essential hypertension is inherent in the development of cardiovascular diseases and advanced CKD is associated with an increased risk of severe COVID-19, optimal treatment/management of hypertension can contribute to a better prognosis of the SARS-CoV-2 infection.

Hence, our systems studies have been mainly focused on analyzing the relationship between taking medications for essential hypertension and the coexistence of COVID-19 disease.

We have found that taking antihypertensive medications, such as angiotensin-converting enzyme inhibitors or angiotensin receptor blockers, does not enhance the development of SARS-CoV-2 infection.

Moreover, when assessing the effectiveness of the treatment of hypertension in the active SARS-CoV-2 infection, we have confirmed the effectiveness of the hypertension treatment in patients medicated by ACEIs. On the other hand, we have observed that the second group of drugs, that is, ARBs, might not properly decrease blood pressure—they may even have slightly the opposite effect.

Additionally, the simultaneous use of ARBs and ACEIs averages the effects of taking both drugs. We have noticed that it leads to an insignificant decrease in blood pressure.

In addition to the results obtained from the study of the relationship between taking antihypertensive medications and the coexistence of COVID-19 disease, we have performed several other analyses that focused on finding elementary processes of crucial importance (high significance) to the functioning of the model of the relation between SARS-CoV-2 infection and essential hypertension. The determined elementary reactions are mainly included in RAA, inflammation and lymphocytes activation, and SARS-CoV-2 infection modules. Moreover, we have defined elementary processes, which are independent of the virus module, that is, the modification of he lymphatic capillary network via VEGFR3, VEGFR3 and VEGFC binding, and the lymphatic endothelium (all of them belong to the endothelial damage module). Furthermore (from the comparative analysis of the results of the significance analysis for the model of essential hypertension with and without the presence of SARS-CoV-2), we have determined which elementary processes are more important for the model with SARS-CoV-2, and which of them are more important in the model without the virus. This comparative analysis has shown that significant differences have been observed only in the two modules, that is, the inflammation and lymphocytes activation module and the RAA module.

The results of these analyses make it possible to determine how the virus might affect the modeled system, which describes a hypothetical patient with essential hypertension and CKD.

## Figures and Tables

**Table 1:** List of transitions of the model with SARS-CoV-2.

ID	Biological Meaning	ID	Biological Meaning
$t_{0}$	neoantigens formation	$t_{44}$	angiotensinogen and renin binding
$t_{1}$	chronic inflammatory process	$t_{45}$	angiotensin I and ACE binding
$t_{2}$	immune system activation via bacterial inflammation	$t_{46}$	ACE synthesis by vascular endothelium in lungs and kidney
$t_{3}$	Th lymphocytes migration into blood vessels	$t_{47}$	decreased in renal perfusion and decreased sodium load in DCT
$t_{4}$	source of cytokines	$t_{48}$	activation of aortic carotid baroreceptors
$t_{5}$	acute phase reaction in liver	$t_{49}$	stimulation of VEGFC formation by bradykinin-B2R complex
$t_{6}$	increased AT1R in vascular smooth muscles cells	$t_{50}$	synthesis and release stimulation
$t_{7}$	AT1R source	$t_{51}$	ACE downregulation
$t_{8}$	T lymphocytes activation and proliferation via AT1R	$t_{52}$	sympathetic stimulation under stress
$t_{9}$	reducing activity of eNOS by CRP	$t_{53}$	AT2R expression
$t_{10}$	osmotically independent binding of Na${}^{+}$ in interstitium	$t_{54}$	RANTES influenced by TNF-TNFR axis
$t_{11}$	immune system activation in interstitium under local hypertonic state	$t_{55}$	shear stress influenced by high blood pressure
$t_{12}$	VEGFC activation	$t_{56}$	convesion of agiotensin II
$t_{13}$	modification of lymphatic capillary network via VEGFR3	$t_{57}$	**S protein and ACE2 binding**
$t_{14}$	NO increase	$t_{58}$	**entering vulnerable cell**
$t_{15}$	induction of Th17	$t_{59}$	**fusing virus membrane with membrane of cell**
$t_{16}$	TonEBP synthesis under local hypertonic state	$t_{60}$	conversion of agiotensin I
$t_{17}$	VEGFR3 and VEGFC binding	$t_{61}$	**SARS-CoV-2 infection**
$t_{18}$	VEGFR2 and VEGFC binding (canonical signalling)	$t_{62}$	Ang-(1-7) binding to Mas receptor induce vasodilation, anti-thrombosis and anti-arrhythmogenic effects
$t_{19}$	muscles relaxation	$t_{63}$	ACE2 synthesis
$t_{20}$	lymphatic endothelium	$t_{64}$	**releasing viral RNA and hijacking cell**
$t_{21}$	vascular endothelial growth factor receptor 2 (VEGFR2) expression	$t_{65}$	conversion
$t_{22}$	TNF-α and TNFR1 binding	$t_{66}$	decreased AT1R
$t_{23}$	TNFR1 expression	$t_{67}$	ANG-(1-9) action
$t_{24}$	endothelial stimulation of ICAM-1, VCAM-1 and PECAM-1	$t_{68}$	**making viral proteins, assembling new copies and spreading infection**
$t_{25}$	vasoconstriction	$t_{69}$	Mas expression
$t_{26}$	lowering blood pressure by PGI2	$t_{70}$	increased PGI2 via ANG-(1-7) and AT2R
$t_{27}$	eNOS expression significantly attenuated by TNF-α	$t_{71}$	stimulation of aldosterone release
$t_{28}$	APC with neoantigens binding	$t_{72}$	increased aldosterone in blood and increased ECF volume
$t_{29}$	blood pressure increasing	$t_{73}$	chronic kidney disease progression
$t_{30}$	eNOS synthesis induction calcium and phosphorylation-dependent	$t_{74}$	MAPK-dependent TACE phosphorylation
$t_{31}$	peroxynitrite (ONOO${}^{-}$) formation	$t_{75}$	proteolytic cleavage of ACE2
$t_{32}$	oxidation of active eNOS cofactor BH${}_{4}$	$t_{76}$	**source of TMPRSS2**
$t_{33}$	superoxide anion radical (O${}_{2}^{•-}$) synthesis by eNOS uncoupling	$t_{77}$	IL-1β action
$t_{34}$	low NO synthesis	$t_{78}$	**immune system activation via viral infection**
$t_{35}$	uncoupled eNOS formation	$t_{79}$	lymphocytes migration
$t_{36}$	NADPH oxidase activation via AT1R	$t_{80}$	enhancement of TNF-α action
$t_{37}$	NADPH oxidase activated via VEGFR2	$t_{81}$	AT1R upregulation
$t_{38}$	non-canonical signalling	$t_{82}$	increased formation of angiotensinogen
$t_{39}$	superoxide anion radical generation via low NO	$t_{83}$	source of lymphocytes
$t_{40}$	superoxide anion radical generation via NADPH oxidase	$t_{84}$	**increased stimulation of IFN-γ via products of viral RNA transcription**
$t_{41}$	superoxide anion radical generation via high blood pressure	$t_{85}$	**increased stimulation of IL-1β via products of viral RNA transcription**
$t_{42}$	ADMA synthesis induction by oxidative stress	$t_{86}$	**increased stimulation of IL-6 via products of viral RNA transcription**
$t_{43}$	angiotensinogen synthesis by liver		

**Table 2:** List of places of the model with SARS-CoV-2.

ID	Biological Meaning	ID	Biological Meaning
$p_{0}$	neoantigens	$p_{28}$	high blood pressure
$p_{1}$	APC cells with neoantigens	$p_{29}$	high coupled eNOS
$p_{2}$	Th1 lymphocytes	$p_{30}$	uncoupled eNOS
$p_{3}$	RANTES and CCR5	$p_{31}$	ADMA
$p_{4}$	activated NADPH oxidase	$p_{32}$	angiotensinogen
$p_{5}$	T lymphocytes in adventitia and perivascular adipose tissue	$p_{33}$	angiotensin I
$p_{6}$	increase in IFN-γ	$p_{34}$	prorenin–renin axis
$p_{7}$	IL-17	$p_{35}$	ACE on vascular endothelium lung and kidney
$p_{8}$	APC (macrophages, B lymphocytes, dendritic cells)	$p_{36}$	ET1
$p_{9}$	CRP	$p_{37}$	TNF-α and TNFR1 complex
$p_{10}$	CD4${}^{+}$ T lymphocytes	$p_{38}$	ACE2 protein functional receptor for SARS-CoV-2 in various human organs
$p_{11}$	ROS (superoxide anion radical (O${}_{2}^{•-}$))	$p_{39}$	ANG-(1-7)
$p_{12}$	Na${}^{+}$ bound to GAGs in interstitium	$p_{40}$	**S protein of SARS-CoV-2**
$p_{13}$	attracted MPS	$p_{41}$	**S protein and ACE2 complex**
$p_{14}$	VEGFC	$p_{42}$	**affected cell**
$p_{15}$	TonEBP	$p_{43}$	ANG-(1-9)
$p_{16}$	VEGFR3	$p_{44}$	**viral RNA**
$p_{17}$	VEGFC-VEGFR3 complex	$p_{45}$	**infected cell after reading RNA**
$p_{18}$	VEGFR2	$p_{46}$	Mas receptor
$p_{19}$	VEGFC-VEGFR2 complex	$p_{47}$	angiotensin II in capillaries
$p_{20}$	TNFR1	$p_{48}$	aldosterone
$p_{21}$	high NO	$p_{49}$	activated TACE
$p_{22}$	high PGI2	$p_{50}$	**TMPRSS2**
$p_{23}$	low NO	$p_{51}$	IL-6
$p_{24}$	AT1R	$p_{52}$	IL-1β
$p_{25}$	AT2R	$p_{53}$	IL-23
$p_{26}$	peroxynitrite (ONOO${}^{-}$)	$p_{54}$	TNF-α
$p_{27}$	decreased blood pressure		

**Table 3:** The results of the significance analysis for selected elementary processes for t-invariants of the model with SARS-CoV-2, which are distinguished into two sets of t-invariants: all t-invariants and only those t-invariants which are related to the SARS-CoV-2 module *.

Modules	Name of Transition	ID	Frequency of Transitions in All t-Invariants of the Base Model with the Virus (139,451 t-inv.)		Occurrence Frequency of Transitions in Selected t-Invariants of the Model with SARS-CoV-2—Only Those Related to the SARS-CoV-2 Module (113,454 t-inv.)	
			Frequency trans./t-inv.		Frequency trans./t-inv.	
(b)	angiotensinogen and renin binding	$t_{44}$	139,409	99.97%	113,431	99.98%
(b)	ACE synthesis by vascular endothelium in lungs and kidney	$t_{46}$	139,401	99.96%	113,431	99.98%
(b)	angiotensin I and ACE binding	$t_{45}$	139,230	99.84%	113,328	99.89%
(c)	acute phase reaction in liver	$t_{5}$	139,048	99.71%	113,314	99.88%
(c)	source of cytokines	$t_{4}$	137,544	98.63%	111,810	98.55%
(e)	MAPK-dependent TACE phosphorylation	$t_{74}$	134,681	96.58%	110,394	97.30%
(g)	osmotically independent binding of Na${}^{+}$ in interstitium	$t_{10}$	133,447	95.69%	109,584	96.59%
(b)	AT2R expression	$t_{53}$	131,074	93.99%	108,040	95.23%
(c)	immune system activation via bacterial inflammation	$t_{2}$	131,032	93.96%	108,026	95.22%
(c)	T lymphocytes activation and proliferation via AT1R	$t_{8}$	131,032	93.96%	108,026	95.22%
(c)	induction of Th17	$t_{15}$	131,032	93.96%	108,026	95.22%
(c)	TNF-α and TNFR1 binding	$t_{22}$	131,032	93.96%	108,026	95.22%
(c)	TNFR1 expression	$t_{23}$	131,032	93.96%	108,026	95.22%
(c)	APC with neoantigens binding	$t_{28}$	131,032	93.96%	108,026	95.22%
(c)	blood pressure increasing	$t_{29}$	131,032	93.96%	108,026	95.22%
(c)	lymphocytes migration	$t_{79}$	131,032	93.96%	108,026	95.22%
(b)	ACE2 synthesis	$t_{63}$	125,129	89.73%	113,454	100.00%
(a)	S protein and ACE2 binding	$t_{57}$	113,454	81.36%	113,454	100.00%
(a)	entering vulnerable cell	$t_{58}$	113,454	81.36%	113,454	100.00%
(a)	fusing virus membrane with membrane of cell	$t_{59}$	113,454	81.36%	113,454	100.00%
(a)	SARS-CoV-2 infection	$t_{61}$	113,454	81.36%	113,454	100.00%
(a)	source of TMPRSS2	$t_{76}$	113,454	81.36%	113,454	100.00%
(d)	vascular endothelial growth factor receptor 2 (VEGFR2) expression	$t_{21}$	106,674	76.50%	86,430	76.18%
(f)	uncoupled eNOS formation	$t_{35}$	94,871	68.03%	77,641	68.43%
(c)	enhancement of TNF-α action	$t_{80}$	91,981	65.96%	77,761	68.54%
(c)	chronic inflammatory process	$t_{1}$	86,157	61.78%	60,287	53.14%
(c)	neoantigens formation	$t_{0}$	84,808	60.82%	70,158	61.84%
(c)	synthesis and release stimulation	$t_{50}$	84,624	60.68%	69,774	61.50%
(f)	NO increase	$t_{14}$	81,628	58.54%	66,484	58.60%
(d)	VEGFR2 and VEGFC binding (canonical signalling)	$t_{18}$	81,628	58.54%	66,484	58.60%
(f)	muscles relaxation	$t_{19}$	81,628	58.54%	66,484	58.60%
(f)	eNOS synthesis induction calcium and phosphorylation-dependent	$t_{30}$	81,628	58.54%	66,484	58.60%
(c)	IL-1β action	$t_{77}$	76,537	54.88%	61,301	54.03%
(b)	AT1R upregulation	$t_{81}$	73,060	52.39%	59,858	52.76%
(b)	increased formation of angiotensinogen	$t_{82}$	69,764	50.03%	56,775	50.04%
(b)	angiotensinogen synthesis by liver	$t_{43}$	69,645	49.94%	56,656	49.94%
(f)	vasoconstriction	$t_{25}$	66,781	47.89%	54,445	47.99%
(e)	NADPH oxidase activation via AT1R	$t_{36}$	65,728	47.13%	54,710	48.22%
(a)	releasing viral RNA and hijacking cell	$t_{64}$	65,517	46.98%	65,517	57.75%
(a)	immune system activation via viral infection	$t_{78}$	65,516	46.98%	65,516	57.75%
(c)	source of lymphocytes	$t_{83}$	65,516	46.98%	42,510	37.47%
(a)	increased stimulation of IFN-γ via products of viral RNA transcription	$t_{84}$	65,426	46.92%	65,426	57.67%

${}^{*}$Table 3 contains only selected transitions for which the significance is above 50% in both the analysis of all t-invariants and t-invariants only associated with the SARS-CoV-2 module.

**Table 4:** Comparison of the significance analysis between the model with SARS-CoV-2 and without the virus (139,451 t-inv. vs. 25,997 t-inv.) *.

Biological Process	The Model with SARS-CoV-2 Infection (139,451 t-inv.)		The Model without SARS-CoV-2 Infection (25,997 t-inv.)		Difference in p.p.
	Frequency trans./t-inv.		Frequency trans./t-inv.		
SARS infection and related subprocesses					
S protein and ACE2 binding	113,454	81.36%	KNOCKOUT		
entering vulnerable cell	113,454	81.36%			
fusing virus membrane with membrane of cell	113,454	81.36%			
SARS-CoV-2 infection	113,454	81.36%			
releasing viral RNA and hijacking cell	65,517	46.98%			
making viral proteins, assembling new copies and spreading infection	1	0.00%			
source of TMPRSS2	113,454	81.36%			
immune system activation via viral infection	65,516	46.98%			
increased stimulation of IFNγ via products of viral RNA transcription	65,426	46.92%			
increased stimulation of IL-1β via products of viral RNA transcription	9503	6.81 %			
increased stimulation of IL-6 via products of viral RNA transcription	33,572	24.07%			
Chronic kidney disease					
chronic kidney disease progression	62,887	45.10%	11,258	43.30%	1.79
Oxidative stress					
peroxynitrite (ONOO${}^{-}$) formation	7696	5.52%	1,386	5.33%	0.19
oxidation of active eNOS cofactor BH${}_{4}$	7696	5.52%	1,386	5.33%	0.19
superoxide anion radical (O${}_{2}^{•-}$) synthesis by eNOS uncoupling	52,323	37.52%	9547	36.72%	0.80
NADPH oxidase activation via AT1R	65,728	47.13%	11,018	42.38%	4.75
NADPH oxidase activated via VEGFR2	53,530	38.39%	10,542	40.55%	−2.16
superoxide anion radical generation via low NO	49,735	35.66%	8942	34.40%	1.27
superoxide anion radical generation via NADPH oxidase	42,986	30.83%	7936	30.53%	0.30
superoxide anion radical generation via high blood pressure	52,626	37.74%	10,370	39.89%	−2.15
MAPK-dependent TACE phosphorylation	134,681	96.58%	24,287	93.42%	3.16
Inflammation and lymphocytes activation					
neoantigens formation	84,808	60.82%	14,650	56.35%	4.46
chronic inflammatory process	86,157	61.78%	25,870	99.51%	−37.73
immune system activation via bacterial inflammation	131,032	93.96%	23,006	88.49%	5.47
Th lymphocytes migration into blood vessels	19,180	13.75%	5070	19.50%	−5.75
source of cytokines	137,544	98.63%	25,734	98.99%	−0.36
acute phase reaction in liver	139,048	99.71%	25,734	98.99%	0.72
T lymphocytes activation and proliferation via AT1R	131,032	93.96%	23,006	88.49%	5.47
reducing activity of eNOS by CRP	34,440	24.70%	6536	25.14%	−0.44
induction of Th17	131,032	93.96%	23,006	88.49%	5.47
TNF-α and TNFR1 binding	131,032	93.96%	23,006	88.49%	5.47
TNFR1 expression	131,032	93.96%	23,006	88.49%	5.47
endothelial stimulation of ICAM-1, VCAM-1 and PECAM-1	18,972	13.60%	4966	19.10%	−5.50
APC with neoantigens binding	131,032	93.96%	23,006	88.49%	5.47
blood pressure increasing	131,032	93.96%	23,006	88.49%	5.47
synthesis and release stimulation	84,624	60.68%	14,850	57.12%	3.56
**Biological Process**	**Frequency trans./t-inv.**		**Frequency trans./t-inv.**		**Difference** **in p.p**.
RANTES influenced by TNF-TNFR axis	19,180	13.75%	5070	19.50%	−5.75
IL-1β action	76,537	54.88%	15,236	58.61%	−3.72
lymphocytes migration	131,032	93.96%	23,006	88.49%	5.47
enhancement of TNF-α action	91,981	65.96%	14,220	54.70%	11.26
source of lymphocytes	65,516	46.98%	23,006	88.49%	−41.51
Nitric oxide					
NO increase	81,628	58.54%	15,144	58.25%	0.28
muscles relaxation	81,628	58.54%	15,144	58.25%	0.28
vasoconstriction	66,781	47.89%	12,336	47.45%	0.44
eNOS expression significantly attenuated by TNF-α	9938	7.13%	1763	6.78%	0.34
eNOS synthesis induction calcium and phosphorylation-dependent	81,628	58.54%	15,144	58.25%	0.28
low NO synthesis	49,796	35.71%	8929	34.35%	1.36
uncoupled eNOS formation	94,871	68.03%	17,230	66.28%	1.75
ADMA synthesis induction by oxidative stress	47,978	34.40%	8660	33.31%	1.09
Endothelial dysfunction					
VEGFC activation	40,815	29.27%	7573	29.13%	0.14
modification of lymphatic capillary network via VEGFR3	2	0.00%	2	0.01%	−0.01
VEGFR3 and VEGFC binding	2	0.00%	2	0.01%	−0.01
VEGFR2 and VEGFC binding (canonical signalling)	81,628	58.54%	15,144	58.25%	0.28
lymphatic endothelium	2	0.00%	2	0.01%	−0.01
vascular endothelial growth factor receptor 2 (VEGFR2) expression	106,674	76.50%	20,244	77.87%	−1.37
lowering blood pressure by PGI2	42	0.03%	28	0.11%	−0.08
non-canonical signalling	12,021	8.62%	2,723	10.47%	−1.85
stimulation of VEGFC formation by bradykinin-B2R complex	40,815	29.27%	7,573	29.13%	0.14
shear stress influenced by high blood pressure	58,160	41.71%	10,520	40.47%	1.24
Extra-renal sodium retention					
osmotically independent binding of Na${}^{+}$ in interstitium	133,447	95.69%	23,863	91.79%	3.90
immune system activation in interstitium under local hypertonic state	40,815	29.27%	7573	29.13%	0.14
TonEBP synthesis under local hypertonic state	40,815	29.27%	7573	29.13%	0.14
RAA					
increased AT1R in vascular smooth muscles cells	53,444	38.32%	11,962	46.01%	−7.69
AT1R source	31,067	22.28%	7136	27.45%	−5.17
angiotensinogen synthesis by liver	69,645	49.94%	12,989	49.96%	−0.02
angiotensinogen and renin binding	139,409	99.97%	25,978	99.93%	0.04
angiotensin I and ACE binding	139,230	99.84%	25,902	99.63%	0.21
ACE synthesis by vascular endothelium in lungs and kidney	139,401	99.96%	25,970	99.90%	0.07
decreased in renal perfusion and decreased sodium load in DCT	59,507	42.67%	11,100	42.70%	−0.02
activation of aortic carotid baroreceptors	59,507	42.67%	11,100	42.70%	−0.02
ACE downregulation	55,806	40.02%	15,432	59.36%	−19.34
sympathetic stimulation under stress	20,419	14.64%	3794	14.59%	0.05
AT2R expression	131,074	93.99%	23,034	88.60%	5.39
**Biological Process**	**Frequency trans./t-inv.**		**Frequency trans./t-inv.**		**Difference** **in p.p**.
conversion of agiotensin II	45,995	32.98%	8372	32.20%	0.78
conversion of agiotensin I	8681	6.23%	1364	5.25 %	0.98
ANG-(1-7) binding to Mas receptor induces vasodilation, anti-thrombosis and anti-arrhythmogenic effects	37,380	26.81%	7052	27.13%	−0.32
ACE2 synthesis	125,129	89.73%	11,675	44.91%	44.82
conversion	8657	6.21%	1348	5.19 %	1.02
decreased AT1R	20,676	14.83%	3168	12.19%	2.64
ANG-(1-9) action	24	0.02%	16	0.06%	−0.04
Mas expression	37,380	26.81%	7052	27.13%	−0.32
increased PGI2 via ANG-(1-7) and AT2R	42	0.03%	28	0.11%	−0.08
stimulation of aldosterone release	28,968	20.77%	5568	21.42%	−0.64
increased aldosterone in blood and increased ECF volume	28,968	20.77%	5568	21.42%	−0.64
proteolytic cleavage of ACE2	12,021	8.62%	2723	10.47%	−1.85
AT1R upregulation	73,060	52.39%	13,202	50.78%	1.61
increased formation of angiotensinogen	69,764	50.03%	12,989	49.96%	0.06

* As significant differences assumed: positive differences greater ≥ 5 p.p., while negative differences ≤ −5 p.p. The interpretation of the “difference in p.p.” column is shown in Figure 4.

**Table 5:** Summary of the most important differences obtained during the comparative analysis (139,451 t-inv. vs. 25,997 t-inv.) *.

Biological Modules	Biological Process	The Model with SARS-CoV-2 Infection (139,451 t-inv.)		The Model without SARS-CoV-2 Infection (25,997 t-inv.)		Difference in p.p.
		Frequency trans./t-inv.		Frequency trans./t-inv.		
(b)	ACE2 synthesis	125,129	89.73%	11,675	44.91%	44.82
(c)	enhancement of TNF-α action	91,981	65.96%	14,220	54.70%	11.26
(c)	immune system activation via bacterial inflammation	131,032	93.96%	23,006	88.49%	5.47
(c)	T lymphocytes activation and proliferation via AT1R	131,032	93.96%	23,006	88.49%	5.47
(c)	induction of Th17	131,032	93.96%	23,006	88.49%	5.47
(c)	TNF-α and TNFR1 binding	131,032	93.96%	23,006	88.49%	5.47
(c)	TNFR1 expression	131,032	93.96%	23,006	88.49%	5.47
(c)	APC with neoantigens binding	131,032	93.96%	23,006	88.49%	5.47
(c)	blood pressure increasing	131,032	93.96%	23,006	88.49%	5.47
(c)	lymphocytes migration	131,032	93.96%	23,006	88.49%	5.47
(b)	AT2R expression	131,074	93.99%	23,034	88.60%	5.39
(b)	AT1R source	31,067	22.28%	7136	27.45%	−5.17
(c)	endothelial stimulation of ICAM-1, VCAM-1 and PECAM-1	18,972	13.60%	4966	19.10%	−5.50
(c)	Th lymphocytes migration into blood vessels	19,180	13.75%	5070	19.50%	−5.75
(c)	RANTES influenced byTNF-TNFR axis	19,180	13.75%	5070	19.50%	−5.75
(b)	increased AT1R in vascular smooth muscles cells	53,444	38.32%	11,962	46.01%	−7.69
(b)	ACE downregulation	55,806	40.02%	15,432	59.36%	−19.34
(c)	chronic inflammatory process	86,157	61.78%	25,870	99.51%	−37.73
(c)	source of lymphocytes	65,516	46.98%	23,006	88.49%	−41.51

${}^{*}$ 5 percentage points (p.p.) has been considered as a significant difference. The interpretation of the “difference in p.p.” column is shown in Figure 4.

**Table 6:** List of non-trivial MCT sets for the model with SARS-CoV-2 (for all t-invariants).

MCT Set	Contained Transitions	Biological Meaning
$m_{1}$	$t_{2}$, $t_{8}$, $t_{15}$, $t_{22}$, $t_{23}$, $t_{28}$, $t_{29}$, $t_{79}$	Immune system’s response to inflammation
$m_{2}$	$t_{57}$, $t_{58}$, $t_{59}$, $t_{61}$, $t_{76}$	The process of SARS-CoV-2 entering the host cells
$m_{3}$	$t_{14}$, $t_{18}$, $t_{19}$, $t_{30}$	Increased level of nitric oxide leads to a decrease in blood pressure
$m_{4}$	$t_{11}$, $t_{12}$, $t_{16}$	Extra-renal sodium retention
$m_{5}$	$t_{13}$, $t_{17}$, $t_{20}$	Modification lymphatic capillary network via VEGFR3
$m_{6}$	$t_{3}$, $t_{54}$	Th lymphocytes migration into the blood vessels
$m_{7}$	$t_{26}$, $t_{70}$	Increased level of PGI2 by ANG-(1-7) and AT2R leads to a decrease in blood pressure
$m_{8}$	$t_{31}$, $t_{32}$	eNOS uncoupling by peroxynitrite anion (ONOO${}^{-}$)
$m_{9}$	$t_{62}$, $t_{69}$	ANG-(1-7) binding to Mas receptor causes vasodilation, anti-thrombosis and anti-arrhythmogenic action
$m_{10}$	$t_{71}$, $t_{72}$	Stimulation of aldosterone release, which leads to an increase in blood pressure

## Data Availability

Not applicable.

## Supplementary Materials

The following are available online at https://www.mdpi.com/article/10.3390/ijms221910518/s1.

## Abbreviations

ACE	angiotensin-converting enzyme
ACE2	angiotensin-converting enzyme 2
ACEI	angiotensin-converting enzyme inhibitor
ADMA	asymmetric dimethylarginine
ANG-(1-9)	angiotensin 1-9
ANG-(1-7)	angiotensin 1-7
APC	antigen-presenting cell
ARB	angiotensin II receptor blockers
AT1R	angiotensin type 1 receptor
AT2R	angiotensin type 2 receptor
B2R	bradykinin B2 receptor
BH${}_{4}$	tetrahydrobiopterin
CCR5	receptor for RANTES
CKD	chronic kidney disease
COVID-19	coronavirus disease of 2019
CRP	C-reactive protein
DCT	distal convoluted tubule
ECF	extracellular fluid
eNOS	endothelial nitric oxide synthase
ET1	endothelin 1
GAGs	glycosaminoglycans
ICAM-1	intercellular adhesion molecule-1
IL-6	interleukin-6
IL-1β	interleukin-1β
IL-17	interleukin-17
IL-23	interleukin-23
IFN-γ	interferon gamma
MAPK	mitogen-activated protein kinase
MCT sets	Maximal Common Transition sets
MPS	mononuclear phagocyte system cells
NO	nitric oxide
O${}_{2}^{•-}$	superoxide anion radical
ONOO${}^{-}$	peroxynitrite
PECAM-1	platelet endothelial cell adhesion molecule-1
PGI2	prostacyclin
RANTES	regulated on activation, normal T-cell expressed and secreted
RAS, RAA	renin-angiotensin-aldosterone system
ROS	reactive oxygen species
SARS-CoV-2	severe acute respiratory syndrome coronavirus-2
TACE	TNF-α (tumour necrosis factor α)-converting enzyme
Th17	T helper 17 cell
TMPRSS2	transmembrane protease, serine 2
TNF-α	tumor necrosis factor alpha
TNFR1	tumor necrosis factor receptor 1
TonEBP	tonicity enhancer binding protein
VCAM-1	vascular cell adhesion molecule-1
VEGF-C	vascular endothelial growth factor C
VEGFR2	vascular endothelial growth factor receptor 2
VEGFR3	vascular endothelial growth factor receptor 3
